# On Delivery in Certain Difficult Cases of Arm-Presentation

**Published:** 1802-06

**Authors:** John Sims

**Affiliations:** Upper Guilford Street


Medical and Phyfical Journal.

VOL. VII.] JUNE, 1802. [NO. XL.

### *On Delivery in certain difficult Cases of Arm-presentation; by* JOHN SIMS, M. D.

IT has been confidered as a general rule in the practice of Midwifery, that, when the arm prefents, except the head is at the fame time defcending into the pelvis, the child muft be turned and brought by the feet; and for a long time it was fuppofed that a full grown child, in fuch a fituation, could never by any effect of the labour-pains be expelled; but Dr. Denman firft obferved that there were exceptions to this, and that a child prefenting with the arm, might, by the long-continued action of the uterus, be fo turned about, that the breech fhould come down, and thus the delivery be completed without affiftance from art. There is no doubt but that this fpontaneous evolution has fometimes really taken place; experience has however fhewn, that it is by no means to be depended upon, and the rule of the neceffity of turning a child prefenting with the arm is generally acted upon, and I believe with great propriety, in thofe cafes where the practitioner is in attendance fufficiently early after the membranes have been ruptured, to allow of the operation of turning being performed with tolerable eafe. But it fometimes happens that from neglect in the beginning,* the child is fuffered to remain in this unfavourable pofition fo long after the difcharge of the liquor amnii, that the uterus contracts fo ftrongly round the body of the child, that the operation of turning becomes extremely difficult, and cannot be performed without ufing fo much force as will not unfrequently prove even fatal to the mother. Every experienced practitioner knows that there are cafes of this kind, in which the difficulty of turning is extreme; and although long-continued efforts, dexteroufly applied, will generally in the end fucceed, yet the event

* It muft be confeffed, that although neglect in the beginning is the moft ufual caufe of thefe difficult labours, it will fometimes happen that from the rupture of the membranes before the commencement of labour, the enfuing difficulty muft be in great meafure unavoidable.

event is ſo often unfavourable, that theſe forcible deliveries are always to be dreaded; and the more experience a man has, the more he wiſhes to avoid the neceſſity of turning under theſe circumſtances.

It will be remembered that I am ſpeaking only of ſuch caſes where this operation cannot be performed without great violence, for it ſometimes happens even when the waters have been very long diſcharged, that the uterus acts with ſo little force that the turning may be effected with tolerable eaſe and great ſafety to the patient.

In the caſes I am ſpeaking of, the previous death of the child is for the moſt part certain from the concomitant circumſtances; the practitioner has therefore nothing to conſider but how he can bring it away with the leaſt pain and hazard to the mother. Many years ago, in a converſation with ſeveral experienced practitioners on this difficulty, I remember to have heard Dr. Garthſhore recommend the ſevering the head from the body of the child, which he repreſented as having eaſily performed with the aſſiſtance of the blunt hook only; an operation mentioned by Heiſter, as newly invented in his time by one Horne, and long before by Celſus*. Dr. Denman, in his excellent Introduction to the Practice of Midwifery, ſpeaks of this mode of delivery; but not having had any experience of it himſelf, paſſes it over ſlightly. Soon after the above-mentioned converſation I was called to a woman, who had been ſeveral days in labour with an arm-preſentation. She had been from the firſt attended by a gentleman advanced in life, but of little experience in this branch of his profeſſion, having ſpent moſt of his time as a ſurgeon in the navy. Having been a pupil of Dr. Wm. Hunter, he had imbibed a notion that Nature, if unmoleſted, was in every caſe adequate to the delivery of the child; he had not of courſe, as he aſſured me, attempted to give any manual aſſiſtance. This caſe was therefore favourable for the ſpontaneous evolution

* *Heiſter, Cap.* 153. *Sect.* 9. "Si vero transverſus eſt, neque dirigi potuit, uncus alæ injiciendus paulatimq; attrahendus eſt. Sub quo fere cervix replicatur retroque caput ad reliquum corpus ſpectat. Remedio eſt *cervix præciſa* ut ſeparatim utraq; pars auferatur. Id unco fit qui, priori ſimilis, in interiori tantum parte per totam aciem exacuitur. Tum id agendum eſt, ut ante caput, deinde reliqua pars auferatur: quia fere majore parte extracta caput in vacuam vulvam prolabitur extrahique ſine ſummo periculo non poteſt." Celſus, lib. 7, c. 29.

This direction to take away the head firſt and afterwards the reſt of the body is hardly practicable, and from what I have ſeen appears to be unneceſſary; the difficulty and danger of delivering the head, always indeed proportionable to the deformity of the bones of the pelvis, being greater in imagination than in reality.

evolution to have taken place, but unfortunately nothing of the kind happened.

When my aſſiſtance was demanded I found the woman apparently dying, the arm and ſhoulder of the child intirely without the os externum, and the uterus ſo cloſely contracted round its body, that having no hopes of ſaving the life of the mother, any attempt at turning was intirely out of the queſtion; yet being deſirous of finiſhing the delivery, for the ſake of the women in attendance and the relatives of the patient, who are greatly more ſhocked at a woman's dying undelivered, than under any other circumſtances, I thought it a good occaſion to put in practice the operation recommended by Dr. Garthſhore, and accordingly paſſed a blunt hook round the neck of the child, which was ſo low down as to be eaſily got at, and pulled forcibly, twiſting at the ſame time with a view of ſeparating the head from the body; but although the child was very putrid, the neck reſiſted a very conſiderable force ſufficient to extract the child double as it was coming down, the head and thorax paſſing at the ſame time. Since this I have often been conſulted in caſes of arm-preſentation, where the waters had been long diſcharged, and the uterus in conſequence very cloſely contracted round the body of the child; in ſome of theſe caſes, long continued efforts have at length ſucceeded in turning the child, but too frequently the event has been fatal; ſometimes the uterus has been ruptured in the operation, and ſometimes where this misfortune has not been known to have happened, the uterus has ſuffered ſo much that fever and death have enſued. The more experience I have had, the more I have been deſirous of rather bringing away the child in any way I could, than running the riſque of theſe very difficult turnings. Sometimes I have been able to get at the head, and with the perforator and crotchet have accompliſhed the delivery; ſometimes calling to mind the ſpontaneous evolution of Dr. Denman, I have ſucceeded in imitating it by fixing the crotchet in the anus or groin, and now and then pulling by the arm, to get the thorax as low down as poſſible; I have perforated this, and getting away piecemeal whatever part preſented, have divided the body in two parts; in this way aukwardly imitating Dr. Garthſhore's operation; or paſſing the crotchet through the thorax and abdomen, have fixed it in the bones of the pelvis, and thus brought the breech down.

Having been lately called, in conſultation with my friend and colleague Dr. Squire, in a caſe where the arm and navel-ſtring preſented, and the labour, under the management of a midwife, having been ſuffered to go on ſome days after the evacuation of the liquor amnii, the uterus was ſo firmly contracted round round the body of the child, that turning could not but with the greateſt difficulty have been accompliſhed. The Doctor having preciſely the ſame ideas of the danger of turning in this caſe from the reſiſtance the uterus gave to his firſt attempts, and the ſame experience of the greater ſafety of delivering by other means, we determined if poſſible to ſave the patient the danger of this operation; and having with ſome difficulty got at the neck of the child, we fixed a crotchet upon it, and guarding the point with the fingers on the oppoſite ſide of the neck, ſucceeded by degrees in ſeparating the head; then taking hold of the arm, the body paſſed with the greateſt eaſe, leaving the head behind, but ſo low down in the pelvis that it was eaſily extracted by a finger in the mouth, and thumb under the chin, without the uſe of any inſtrument, or the aſſiſtance of a labour pain. The child was not large, or the difficulty of ſeparating the vertebræ of the neck by means of the crotchet would have been greater; it may however, I apprehend, always be accompliſhed with this inſtrument much eaſier than with the common blunt hook; but if filed to an edge on the inner ſide of the bend, this might perhaps be a very convenient inſtrument for ſuch a purpoſe, and appears to be the very ſame as that recommended by Celſus.

As I do not know that any Caſe has been publiſhed in which the practice of Horne, as recommended by Heiſter, and ſince recommended by Dr. Garthſhore, has been had recourſe to, and the event being fully anſwerable to our expectations, I preſume it will be thought worthy to be inſerted in the Medical Journal.

In the above account I have purpoſely ſpoken of the uterus as being ſtrongly contracted round the body of the child, without adverting to the nature of that contraction, as ariſing from the natural elaſticity of the uterus, or from it's increaſed action in expulſive efforts or labour-pains; becauſe, whatever be the kind of contraction, the nature of the difficulty is the ſame, varying only in degree. It may not be amiſs, however, to repeat here what has been properly laid down by Dr. Denman, that whenever there are ſtrong labour-pains preſent, turning ought not to be attempted, and that not only on account of the increaſed difficulty and danger of the operation, but becauſe whenever there are ſtrong forcing pains preſent, a favourable iſſue may under all circumſtances be expected; for as if Nature felt her own inability to accompliſh the delivery in croſs-preſentations, there are rarely any pains; and where theſe exiſt to a certain degree, I have always found that from the ſmall ſize of the child, or, which amounts to the ſame, the large dimenſions of the pelvis, the child was about to be expelled double, or that the head or breech were coming down with the arm, though not yet within within reach of the finger. Often, however, when there are no pains, whilft the patient is left to herfelf, the uterus is immediately thrown into ftrong action by the irritation which the attempt to introduce the hand muft neceffarily occafion, and this circumftance always adds to the difficulty and danger of the operation. It feems probable that opium, fo frequently recommended to be given before the operation is commenced, may in this cafe be ufeful; I have not however been able to decide whether it is really fo or not. Opium feems to me to increafe the action of the uterus as exerted in labour-pains, although its irregular fpafmodic action, producing pains refembling thofe of labour, is certainly diminifhed by it. But notwithftanding this irritability of the uterus, by proceeding with caution in the manner recommended by Dr. Denman, carefully avoiding all exertion of force during the action of the womb, the delivery may for the moft part be fafely accomplifhed, though with great trouble to the operator; for by repeatedly exciting the action of the uterus, its contracting powers become more and more languid, and at length frequently ceafe entirely, when the delivery may be accomplifhed with tolerable eafe and fafety. If, on the other hand, a ftrong man is determined, without regard to the refiftance, to overcome every obftacle, he may fucceed in his purpofe, and that perhaps in a little time, but the event will be too frequently fatal.

JOHN SIMS.

*Upper Guilford Street, May* 1, 1802.

